# Chloroplastic and nuclear diversity of wild beets at a large geographical scale: Insights into the evolutionary history of the *Beta* section

**DOI:** 10.1002/ece3.3774

**Published:** 2018-02-14

**Authors:** Pascal Touzet, Sarah Villain, Laetitia Buret, Hélène Martin, Anne‐Catherine Holl, Céline Poux, Joël Cuguen

**Affiliations:** ^1^ Univ. Lille CNRS, UMR 8198 – Evo‐Eco‐Paleo Lille France

**Keywords:** allo‐polyploidy, *Beta* genus, mating systems, phylogeny

## Abstract

Historical demographic processes and mating systems are believed to be major factors in the shaping of the intraspecies genetic diversity of plants. Among Caryophyllales, the *Beta* section of the genus *Beta,* within the Amaranthaceae/Chenopodiaceae alliance, is an interesting study model with species and subspecies (*Beta macrocarpa*,* Beta patula*,* Beta vulgaris maritima* and *B.v. adanensis)* differing in geographical distribution and mating system. In addition, one of the species, *B. macrocarpa*, mainly diploid, varies in its level of ploidy with a tetraploid cytotype described in the Canary Islands and in Portugal. In this study, we analyzed the nucleotide diversity of chloroplastic and nuclear sequences on a representative sampling of species and subspecies of the *Beta* section (except *B. patula*). Our objectives were (1) to assess their genetic relationships through phylogenetic and multivariate analyses, (2) relate their genetic diversity to their mating system, and (3) reconsider the ploidy status and the origin of the Canarian *Beta macrocarpa*.

## 1 INTRODUCTION

The nature of forces that shape genetic diversity of species is a long‐standing question in evolutionary biology (Leffler et al., 2012). Both historical demographic process that occurred during glaciation periods and life history traits are generally admitted to be the major factors influencing the present intraspecies genetic diversity. In plants, mating systems are believed to be of main importance, in particular the frequent transition to self‐fertility that is expected to affect both neutral diversity and the efficacy of selection (Glémin, 2007; Glémin, Bazin, & Charlesworth, 2006). Empirical studies in a set of species have partially confirmed these theoretical expectations (reviewed in Glémin & Galtier, 2012 and in Castric, Billiard, & Vekemans, 2013). Another evolutionary mechanism influencing plant species diversification is polyploidization. This can occur after interspecies hybridization (allo‐polyploidy) or intraspecific genome duplication (autopolyploidy) (reviewed by Soltis, Marchant, Van de Peer, & Soltis, 2015). It has been generally believed that allopolyploids are more frequent than autopolyploids thanks to the expected gain in fitness of hybrids combining two diverged genomes and thus enlarging their ability of conquering new environments (Abbott et al., 2013). However, autopolyploid occurrence seems to have been underestimated as it appears to be as frequent as allopolyploids, partly due to the difficulty in phenotypically distinguishing them from their diploid counterparts (Barker, Arrigo, Baniaga, Li, & Levin, 2016).

The Betoideae constitute a small subfamily of the Amaranthaceae/Chenopodiaceae alliance that is characterized by a unique fruit type, a capsule that normally opens with a circumscissile lid (Kadereit, Hohmann, & Kadereit, 2006). Within this subfamily, two groups have been defined: Hablitzieae and Beteae which is composed by a single genus, *Beta*. This genus is partitioned in two sections: sect. *Corollinae* (including the previous section *Nanae*) and sect. *Beta* (see Biancardi, Panella, & Lewellen, 2012 for the recent evolution of *Beta* taxonomy). This last section is composed of *B. macrocarpa*,* B. patula* and the species complex *B. vulgaris*, within which can be found wild forms (*B. v. maritima*,* B. v. adanensis*), cultivars (*B. v. vulgaris)* and weeds, a hybrid between *B. v. maritima* and *B. v. vulgaris* (Desplanque et al., 1999). Species of the *Beta* section differ in their respective geographical distribution. *Beta patula* is endemic to two islets of the Madeira Island and one islet at Desertas Islands (Romeiras et al., 2016). *Beta macrocarpa* has been described as two different cytotypes: one diploid cytotype distributed from Portugal to Turkey, along the Mediterranean Basin, and a tetraploid one found in the Canary Islands (Buttler, 1977) and in Portugal (Castro, Romeiras, Castro, Duarte, & Loureiro, 2013). Within *Beta vulgaris*, while *B.v. maritima* populations are found on a large geographical area, along both the Atlantic coasts of Western Europe and most of the Mediterranean coast, *B. v. adanensis* is restricted in the eastern part of the Mediterranean Basin (Aegean islands, Turkey and Syria). In addition, subspecies of the Beta section differ in their mating system: *B.v. maritima* is allogamous and self‐incompatible, while *B. macrocarpa* and *B. v. adanensis* have been described as self‐compatible (Bruun et al., 1995; Letschert, 1993).

This section exhibiting variation in breeding systems, ploidy but also life history traits (Hautekèete, Piquot, & Van Dijk, 2001; Letschert, 1993) is therefore an interesting group to infer their impact on genetic diversity. Former genetic studies have focused on the *B. vulgaris* species complex (Desplanque et al., 1999, 2000; Letschert, 1993; Nishizawa, Kubo, & Mikami, 2000; Nishizawa, Mikami, & Kubo, 2007), or more specifically on *B.v. maritima* as the main genetic resource of cultivated beet (Andrello, Henry, Devaux, Desprez, & Manel, 2016; Andrello et al., 2017; Cuguen et al., 1994; Fénart, Touzet, Arnaud, & Cuguen, 2006; Fievet, Touzet, Arnaud, & Cuguen, 2007; Leys et al., 2014; Raybould, Mogg, & Clarke, 1996; Raybould, Mogg, Gliddon, Thorpe, & Clarke, 1998; Richards, Reeves, Fenwick, & Panella, 2014), while some information is available at the section level (Jung et al., 1991; Shen, Newbury, & Ford‐Loyd, 1996; Letschert, 1993; Hohmann, Kadereit, & Kadereit, 2006; Kadereit et al., 2006; Andrello et al., 2016, 2017; Romeiras et al., 2016). Therefore, in the present study, we analyze the nucleotide diversity of a representative sampling of species and subspecies of the Beta section (except *B. patula*) at chloroplastic and nuclear loci in order to: (1) assess their genetic relationships through phylogenetic and multivariate analyses, (2) relate species/subspecies diversity of the section to their mating system, and (3) reconsider the ploidy status and the origin of the Canarian *Beta macrocarpa*.

## 2 MATERIAL AND METHODS

### 2.1 Plant species and sampling

**Figure 1:**
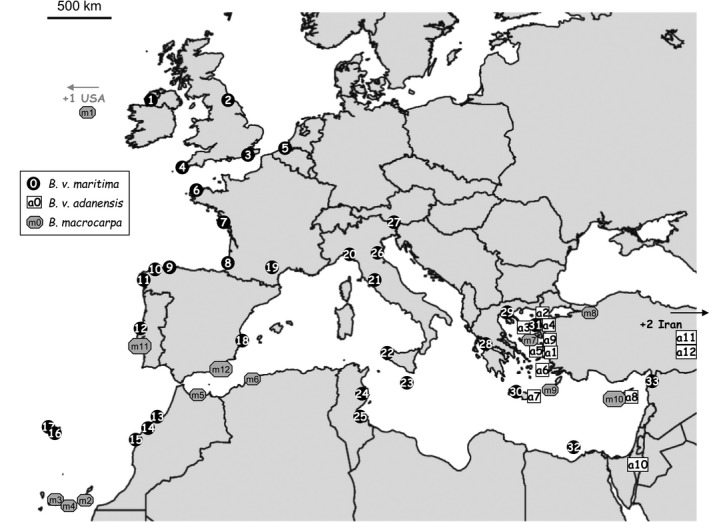
Map of the geographical location of the 57 samples of *Beta*

Seeds from the *Beta* section were obtained from the Federal Centre for Breeding Research on Cultivated Plants of Braunschweig, from the University of Birmingham and from our lab's collection. Details on sampling are given in Table 1. For the study of chloroplastic and nuclear nucleotide diversity, a total of 33 individuals of *Beta v. maritima*, 12 *Beta v. adanensis* and 12 *Beta macrocarpa* were analyzed (Figure 1). These accessions were chosen on the basis of their geographical location. For each location, DNA was extracted from a single individual.

**Table 1:** Localities of samples. The species, the sample numbers, the site of origin (country and location), the IDBBNR accession number (unique identification number assigned to an accession by the Beta International Database) are given, as well as the donor institution: BGRC: Braunschweig Genetic Ressources, Birm.: University of Birmingham, Lille: our lab collection

Species	Sample number	Country	Location	IDBBNR	Donor
*B. v. maritima*	1	Ireland	Sligo	5905	BGRC
	2	Great Britain	Scarborough	5915	BGRC
	3	Great Britain	Ramsgate		Lille
	4	Great Britain	Land's end		Lille
	5	Netherlands	Zwin		Lille
	6	France	Roscoff		Lille
	7	France	Sables d'Olonne		Lille
	8	France	Erromardie		Lille
	9	Spain	Foz		Lille
	10	Spain	Punta Fouxeira		Lille
	11	Spain	Playa de la Lanzada		Lille
	12	Portugal	Obidos	7069	BGRC
	13	Morocco	Casablanca	8550	BGRC
	14	Morocco	Essaouira	8560	BGRC
	15	Morocco	Safi	8556	BGRC
	16	Portugal	Madeira	6069	BGRC
	17	Portugal	Ponto do Parvo		Lille
	18	Spain	Los Arenetes		Lille
	19	France	Bages		Lille
	20	Italy	Fosso d'Arno, Toscana	9452	BGRC
	21	Italy	Lazio	9461	BGRC
	22	Italy	Sicily	2205	BGRC
	23	Malta		8615	BGRC
	24	Tunisia	Sfax	3542	BGRC
	25	Tunisia	Bor. Djilidj	415	BGRC
	26	Italy	Veneto	9481	BGRC
	27	Croatia	Istria	6952	BGRC
	28	Greece	Levkas	139	BGRC
	29	Greece	Khalkidhiki	208	BGRC
	30	Greece	Kissamos, Crete		Lille
	31	Greece	Lesbos		Lille
	32	Egypt	Matruh	9742	BGRC
	33	Turkey	Hatay	8440	BGRC
*B. v. adanensis*	a1	Greece	Samos		Lille
	a2	Turkey	Canakkale	3010	BGRC
	a3	Greece	Lesbos		Lille
	a4	Turkey	Izmir	3016	BGRC
	a5	Greece	Chios		Lille
	a6	Greece	Kos		Lille
	a7	Greece	Kokinos		Lille
	a8	Cyprus	Paphos	7119	BGRC
	a9	Turkey	Aydin	8462	BGRC
	a10	Israel	Zomet Lakhish	3798	BGRC
	a11	Iran	Sorkan, Khouzestan	8623	BGRC
	a12	Iran	Minab, Hormozgan	8622	BGRC
*B. macrocarpa*	m1	United States of America^a^	California	1570	Birm.
	m2	Spain	Fuerteventura	1631	Birm.
	m3	Spain	Tenerife	1571	Birm.
	m4	Spain	Gran Canaria	8569	BGRC
	m5	Morocco	Driouch	8549	BGRC
	m6	Algeria	Mostaganem	1771	Birm.
	m7	Greece	Chios		Lille
	m8	Turkey	Izmit	1188	BGRC
	m9	Greece	Karpathos	6371	BGRC
	m10	Cyprus	Limassol	7127	BGRC
	m11	Portugal	Alcochete	4779	BGRC
	m12	Spain	La Hoya Ruines	2212	BGRC

a Introduced.

Additional samples from the Beta genus, belonging to the *Corollinae* section, were sequenced in order to root the phylogenetic trees: *Beta lomatogona* (PI198401)*, Beta macrorhiza (*BETA 545) (kindly provided by Lothar Freese, Julius Kühn‐Institut, Quedlinburg, Germany) *and Beta nana* (kindly provided by Lee Panella, USDA, Fort Collins, USA).

### 2.2 DNA amplification and sequencing

The DNA extraction from dried leaf tissue was carried out with a Nucleospin^®^96Plant kit (Macherey‐Nagel) on a Microlab^®^Star robot (Hamilton).

#### 2.2.1 cpDNA sequences

Four cpDNA regions were selected for sequencing: the *trnK* intron (K1K2) including the *matK* gene, the *trnD‐trnT* intergenic spacer (DT), the *trnL‐trnF* intergenic spacer (LF), and the 5′ part of the intergenic spacer HK ranging between *trn*H and *psb*A. On account of its size (about 1,900 base pairs [bp]), the K1K2 region was amplified in two overlapping fragments.

The set of primers (forward/reverse) used was 5′‐GTTGCCCGGGATTCGAA‐3′/5′‐ATTAGGGCATCCCATTAGTA‐3′ for the first part of K1K2 (annealing temperature [*T*_a_] = 54°C for the Beta section/58°C for the *Corollinae* section) (modified from Grivet & Petit, 2003) and 5′‐CTAGCACAAGAAAGTCGAAG‐3′/5′‐GGATTTCTAACCATCTTGTT‐3′ for the second part of K1K2 (*T*_a_ = 50°C/58°C); 5′‐ACCAATTGAACTACAATCCC‐3′/5′‐CTACCACTGAGTTAAAAGGG‐3′ for DT (*T*_a_ = 56.5°C/58°C) (Grivet & Petit, 2003); 5′‐GGTTCAAGTCCCTCTATCCC‐3′/5′‐ATTTGAACTGGTGACACGAG‐3′ for LF (*T*_a_ = 57.5°C) (Taberlet et al., 1991); 5′‐CGACCAAAATAACCATGAGC‐3′/5′‐GCTATGCATGGTTCCTTGGT‐3′ for HK (*T*_a_ = 57°C). This last fragment could not be amplified for the 3 *Corollinae* species.

PCR amplification was performed in a 25 μl mix containing 25 ng of DNA template, 3 mmol/L of MgCl_2_, 1.5 μmol/L of Buffer 10X (Perkin‐Elmer, Norwalk, CT, USA), 0.2 μmol/L of each primer, 200 μmol/L of each dNTP, and 0.625 U/μl of hot start *Taq* polymerase (AmpliTaq Gold, Perkin‐Elmer, Norwalk, CT, USA). PCR mixture underwent the following conditions on a 9700 thermal cycler (Perkin‐Elmer, Norwalk, CT, USA): 12‐min denaturing at 94°C, 40 cycles of 30″ denaturing at 94°C, 45″ annealing at *T*_a_ (see above) and from 1 to 2 min extension (depending on the fragment length) at 72°C and a final extension step at 72°C for 10 min, after 40 cycles. The PCR products were then purified using a *QIAquick PCR Purification Kit* (QIAGEN, Inc., Valencia, CA, USA) and directly sequenced with an ABI Prism^™^ BigDye Terminator Cycle Sequencing Ready Reaction Kit (Perkin‐Elmer, Norwalk, CT, USA). Sequence data were obtained on a 3100‐*Avant* Genetic Analyser (Applied Biosystems).

#### 2.2.2 Nuclear DNA sequences

For nuclear analysis, three genes, largely used in phylogenetic studies, were partially sequenced: the alcohol dehydrogenase (*adh*) with primers 5′‐TGTCCTGCCTGTTTTCACTG‐3′/5′‐TACTGCTCCTAGGCCGAAAA‐3′ (*T*_a_ = 61°C/53°C) anchored in exons 1 and 2, the chlorophyll a/b‐binding protein *cab*11 with primers 5′‐CTTCATTAGCTGAGGAACC‐3′/5′‐GCTCTGACATTGGAAACCC‐3′ (*T*_a_ = 55°C) anchored in exons 1 and 2, and the *ITS* region (internal transcribed spacers ITS1 and ITS2 of nuclear ribosomal DNA and the 5.8S rRNA gene) with primers 5′‐GGAAGTAAAAGTCGTAACAAGG‐3′/5′‐TCCTCCGCTATATGATGC‐3′ (*T*_a_ = 53°C) anchored in ITS1 and ITS2 (White et al., 1990). Both PCR and sequencing were done as described in the cpDNA section.

PCR products were directly sequenced for the autogamous diploid species *B.v adanensis* and *B. macrocarpa* and for the *Corollinae* species. For the outcrossers *B. v. maritima* and tetraploid *B. macrocarpa*, PCR products were cloned into pCR2.1‐TOPO using TOPO TA Cloning Kit (Invitrogen, Carlsbad, CA) before sequencing. A minimum of six clones was sequenced to reliably identify both haplotypes and examine PCR‐generated errors due to nucleotide misincorporation and/or recombination.

All sequences generated in the present study have been registered in Genbank (KP747713–KP748171).

### 2.3 Data analyses

DNA sequences were assembled with SEAVIEW (Gouy, Guindon, & Gascuel, 2010), aligned with MAFFT v.7 (Katoh & Standley, 2013) and manually checked and cleaned using Gblocks (Castresana, 2000) when necessary (*Cab*11). The cpDNA alignment with outgroups (for the phylogenetic reconstruction) or without outgroups (for diversity analyses) displayed a total size of 3742 bp and 3752 bp, respectively (K1K2: 1892 bp/1905 bp, DT: 914 bp/911 bp, LF: 301 bp and HK: 635 bp). For the nuclear alignment, the discrepancy between both alignments (with and without outgroups) is mainly due to *Cab*11 for which the intron could not be aligned between *Beta* and *Corollinae* sections. The *Adh* alignment was 349 bp, the *Cab*11 displayed 797 bp without outgroup and 1,140 bp when outgroups were included (692 bp after removing the poorly aligned sites), and the *ITS* region was 668 bp/674 bp long (without/with outgroup).

#### 2.3.1 Phylogenetic and haplotype network reconstructions

The alignment resulted in a dataset of 3,742 bp for the chloroplastic dataset (K1K2, LF, DT and KH) and of 1715 bp (*adh, cab11* and ITS) for the nuclear alignment. Phylogenetic reconstructions based on both chloroplastic and nuclear concatenated datasets and on each nuclear gene separately were performed by maximum likelihood (ML) with PHYML v.3.0 (Guindon et al., 2010) and by Bayesian analyses with MrBAYES, version 3.2.2 (Ronquist et al., 2012).

For the individual nuclear genes analyses, heterozygotes samples were represented by both alleles. For the concatenated analysis, each individual was represented by only one sequence per gene because alleles from different nuclear loci cannot be phased; heterozygous sites were therefore encoded according to the DNA ambiguity code. However, all alleles from the *Beta macrocarpa* 4× individuals were kept in the concatenated analysis as they obviously were from different origins.

The best fitting model of sequences evolution was selected from the BIC (Bayesian Information Criterion) output of jMODELTEST, version 2.1.3 (Darriba, Taboada, Doallo, & Posada, 2012) for each data partition.

For the Bayesian analysis of the concatenated chloroplastic dataset, four partitions corresponding to the four genes were defined. Similarly for the concatenated nuclear dataset, five partitions were considered: they correspond to the intronic and exonic regions of the *adh* and c*ab11* genes and to ITS. For the ML analyses, datasets, concatenated or not, were considered as one partition.

Analyses with MrBAYES were done as follows: two runs of four Markov chains were calculated simultaneously for 1,000,000 to 5,000,000 generations depending on the dataset, with initial equal probabilities for all trees and a random starting tree. Trees were sampled each 100 generations, and the consensus tree with posterior probabilities (PP) was calculated after removal of the first 25% to 50% (according to the analysis) of the total number of generated trees (according to the analysis). The average standard deviation of split frequencies between the two independent runs was lower than 0.01.

PopART v1.7 (Leigh & Bryant, 2015) was used to construct the chloroplastic haplotype TCS network.

#### 2.3.2 Principal component analysis

In order to assess the existence of genetic clusters within the *Beta* section, we conducted a principal component analysis (PCA) on the concatenated nuclear sequences of all individuals except for the samples *B. v. maritima* 6 and *B. v. adanenis* a10 (*adh* sequence was missing for 6, and *cab11* sequence for a10) using *adegenet* R package (Jombart, 2008; R Core Team Development 2014).

#### 2.3.3 Statistical analyses–nucleotide diversity parameters

For each species/subspecies of the *Beta* section, we estimated the nucleotide diversity both as π, the average number of nucleotide differences per site between a pair of randomly chosen sequences (Nei, 1987), and as Watterson's θ_w_ (Watterson, 1975). Among species/subspecies of the *Beta* section, we calculated shared and fixed polymorphisms and the nucleotide divergence (Dxy), using DnaSP version 5 (Librado & Rozas, 2009).

## 3 RESULTS

### 3.1 Phylogenetic analyses

**Figure 2:**
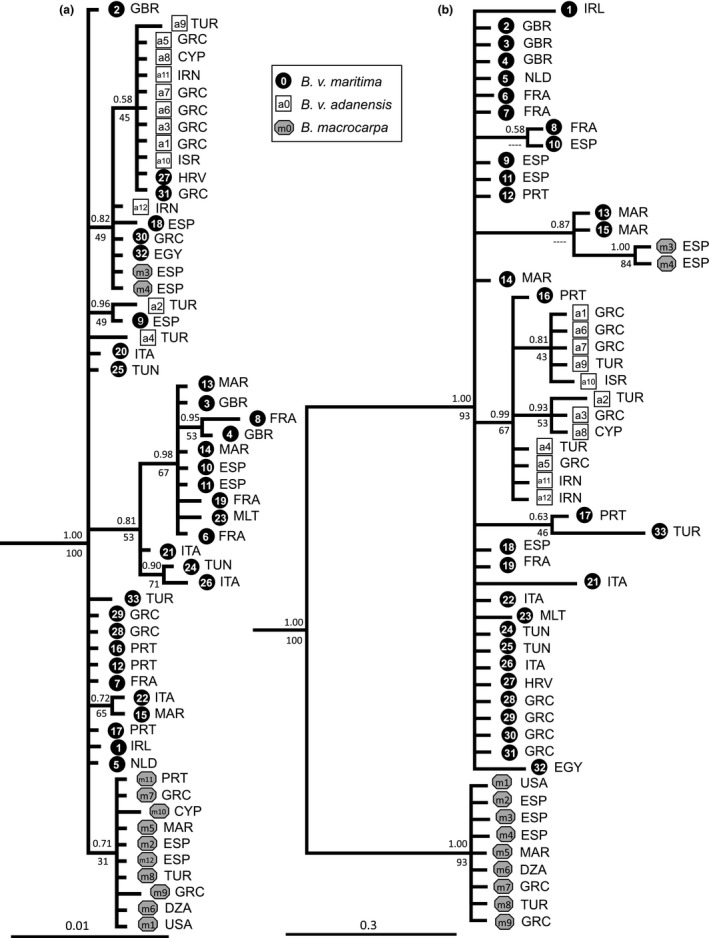
Phylogenetic relationships of Beta section as inferred by Bayesian analyses on the concatenated chloroplastic (a) and concatenated nuclear (b) datasets. The maximum likelihood (ML) analyses resulted in close topologies. Posterior probabilities (PP) and bootstrap percentages (BP) are indicated above and below the branches, respectively. For incongruent nodes between Bayesian and ML topologies, dashes replace BP values. The *Corollinae* species used as outgroup are not shown on the figure for the purpose of clarity. We indicated for each accession the iso‐alpha3 code of the country of origin (Algeria—DZA, Croatia—HRV, Cyprus—CYP, Egypt—EGY, France—FRA, Great Britain—GBR, Greece—GRC, Iran—IRN, Ireland—IRL, Israel—ISR, Italy—ITA, Malta—MLT, Morocco—MAR, Portugal—PRT, Spain—ESP, Netherlands—NLD, Tunisia—TUN, Turkey—TUR, United States of America—USA)

The concatenated chloroplastic sequences from the 57 samples of the *Beta* section and 3 samples from the *Corollinae* section enabled us to generate a rooted phylogenetic tree that revealed (Figure 2a) several clades however with low bootstrap (BP) and posterior probabilities (PP): (1) a clade composed of all *Beta macrocarpa* samples except two samples from the Canary Islands (samples from islands Tenerife and Gran Canaria—m3 and m4), (2) a large clade within which we found most of the *B.v. adanensis* samples and some Eastern *B. v. maritima* samples as well as the two *B. macrocarpa* samples from the Canary Islands, and (iii) a large clade composed mainly of Western *B. v. maritima* samples. The remaining samples were not assigned to a particular clade (see also the haplotype network, Figure S1).

The low bootstrap values reflect the extremely low diversity level displayed by the sequences.

The three nuclear loci sequenced on the same samples (partial sequences of *adh*,* cab11* and the ITS region) led to a nuclear consensus phylogenetic tree (Figure 2b) on which we found this time with a good phylogenetic support: (1) a clear distinct *B. macrocarpa* clade (BP = 93, PP = 1.00) and (2) a large clade composed of *B. v. maritima* and *B. v. adanensis* (BP = 93, PP = 1.00). All *B. v. adanensis* sequences were regrouped in a clade (BP = 67, PP = 0.99) displaying as well one *B. v. maritima* individual.

Notably, only one allele was found for the *B. v. adanensis* and *B. macrocarpa* samples, as expected for autogamous species, whereas two alleles could be found for the allogamous *B. v. maritima* samples. Two alleles per individual were also found for the two Canarian *B. macrocarpa* individuals (m3 and m4) with one allele belonging to the *B. macrocarpa* clade and the other to the *B. v. maritima* clade. These two individuals are most likely tetraploid, resulting from the hybridization between *B. v. maritima* and *B. macrocarpa*. One‐locus trees can be found in the supplementary data (Figures S2–S4).

Overall, chloroplastic and nuclear phylogenetic trees showed that (1) within the *B. vulgaris* species, *B. v. maritima* exhibited the largest diversity, while *B. v. adanensis* represented a sublineage within the *B. v. maritima clade*, (2) the *B. macrocarpa* samples formed a distinct monophyletic lineage from the *B. vulgaris* subspecies (except for the distinctive m3 and m4 samples), and (3) the two *B. macrocarpa* samples from the Canary Islands (m3 and m4) displayed a *B. v. maritima* chloroplastic haplotype and exhibit a hybrid pattern with two nuclear alleles, one maritima‐like and one macrocarpa‐like (Figure 2a,b).

### 3.2 Principal component analysis

**Figure 3:**
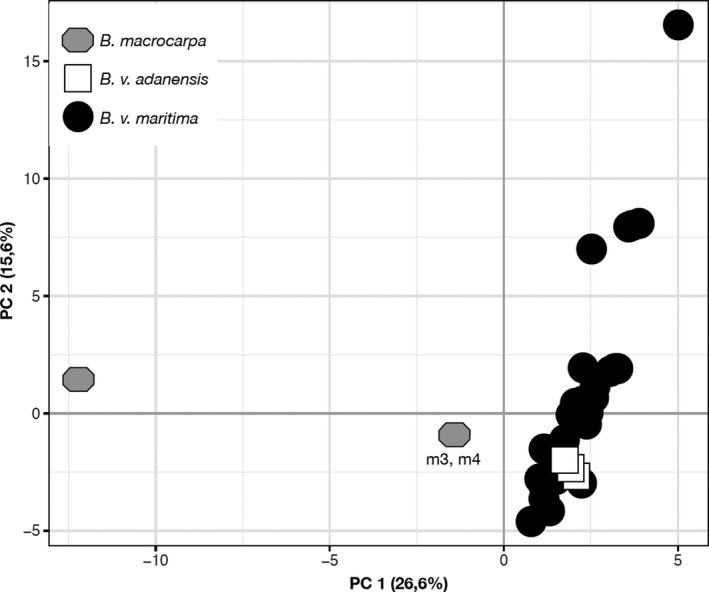
Principal component analysis based on the concatenated nuclear sequences. *B.v. maritima*,* B.v. adanensis*, and *B. macrocarpa* are distinguished by shapes and colors (black, white, and gray, respectively). All *B. macrocarpa* are represented by one unique spot (since they share the same nucleotide sequence) except for m3 and m4 (as indicated)

The principal component analysis (PCA) was consistent with the phylogenies (Figure 3). The first axis of the PCA, representing 26.6% of the variance, separated *B. macrocarpa* from *B. vulgaris* subspecies. As expected by the phylogenetic analysis, the m3 and m4 samples were at an intermediate position between the *B. macrocarpa* cluster and the *B.v. maritima* one, confirming their hybrid status. The diversity of *B. v. maritima* was spread along the second axis that explained 15.6% of the variance, with no clear geographical pattern, while *B.v. adanensis* accessions remained aggregated.

### 3.3 Nucleotide diversity of the *Beta* section

The representative distribution of the sampling enabled us to measure the overall nucleotide diversity of the members of the *Beta* section, at both chloroplastic and nuclear levels (Table 2). At the species/intraspecies level, for both genomes, *B. v. maritima* exhibited the highest level of diversity, followed by *B. v. adanensis* and last *B. macrocarpa* displaying the more conserved sequences. This result was obtained whatever parameter was considered (except for π calculated on *adh*) (Table 2). Note that we did not include in the analyses the two *B. macrocarpa* from Tenerife and Gran Canaria islands since they are most likely allo‐tetraploid. We will call them 4× in the rest of the article for the purpose of clarity.

**Table 2:** Species diversity of the *Beta* section. At each locus, chloroplastic (cp) and nuclear loci (*Adh*,* Cab11*, and ITS) and for each species/subspecies are given: the number of populations per species (Pop) and sequences (Seq), number of haplotypes, number of segregating sites, diversity per site estimated from the total number of mutations (Θ_w_), diversity as the average number of nucleotide differences per site between a pair of randomly chosen sequences (π) with standard deviation (*SD*)

Locus	Species	Pop/Seq	Length (bp)	Number of haplotypes	Segregating sites	Θ_w_ ± *SD* (×10^−3^)	π ± *SD* (×10^−3^)
*Cp*	*B.v. maritima*	33/33	3,752	16	16	1.05 ± 0.40	0.97 ± 0.08
	*B. v. adanensis*	12/12	3,752	5	5	0.44 ± 0.25	0.26 ± 0.09
	*B. macrocarpa*	10/10	3,752	3	2	0.19 ± 0.14	0.11 ± 0.05
*Adh*	*B.v. maritima*	31/62	349	9	8	4.88 ± 2.10	1.26 ± 0.28
	*B. v. adanensis*	12/24	349	2	1	0.77 ± 0.77	1.49 ± 0.09
	*B. macrocarpa*	6/12	349	1	0	0	0
*Cab11*	*B.v. maritima*	32/64	797	25	37	10.53 ± 3.21	9.61 ± 0.53
	*B. v. adanensis*	11/22	797	1	0	0	0
	*B. macrocarpa*	7/14	797	1	0	0	0
*ITS*	*B.v. maritima*	32/64	669	2	3	0.95 ± 0.58	2.28 ± 0.05
	*B. v. adanensis*	12/24	669	2	1	0.40 ± 0.40	0.58 ± 0.14
	*B. macrocarpa*	7/14	669	1	0	0	0

At the chloroplastic level, *B. v. maritima* exhibited 3 times as many haplotypes as *B. v. adanensis* and 5 times as many haplotypes as *B. macrocarpa*. The same pattern was also observed when estimating nucleotide diversity, which differed almost in an order of magnitude between *B. v. maritima* and *B. macrocarpa*.

At the nuclear level, once again, *B. v. maritima* was the most polymorphic species/subspecies at any analysed locus. As previously mentioned, *B. v. adanensis* and *B. macrocarpa* were homozygous for every analysed locus. Note that it was also the case for the two 4× *B. macrocarpa* individuals as we found only one allele for the locus coming from the *B. v. maritima* genome, and one allele for the locus coming from the *B. macrocarpa* genome, suggesting that *4× B. macrocarpa* may preferentially reproduce by selfing.

For *B.v. maritima*,* cab11* was the most polymorphic locus with 25 segregating alleles, then *adh* with 9 alleles, and then *ITS* with only 2 alleles. For *B. v. adanensis*, polymorphism was reduced with two alleles on *adh* and *ITS*, and only one allele on *cab11*. Strikingly, *B. macrocarpa* was fixed on each analysed locus despite the large geographical distribution of the species sample.

In parallel of the phylogenetic and PCA analyses, the level of divergence between the members of the *Beta* section can be described by assessing the number of private and shared polymorphisms among members, as well as the number of fixed differences (Table 3).

**Table 3:** The number of species‐specific polymorphisms, shared polymorphisms, fixed differences, and nucleotide divergence (Dxy) (Jukes‐Cantor) between *Beta* species

Locus	Species comparison (species1/species2)	Species1 only	Species2 only	Shared	Fixed	Dxy ± *SD* (×10^−3^)
*Cp*	*Maritima/adanensis*	13	2	3	0	1.04 ± 0.22
	*Maritima/macrocarpa*	15	1	1	1	1.21 ± 0.25
	*Adanensis/macrocarpa*	4	1	1	2	1.07 ± 0.33
*Adh*	*Maritima/adanensis*	8	1	0	0	4.68 ± 0.91
	*Maritima/macrocarpa*	8	0	0	2	8.88 ± 2.23
	*Adanensis/macrocarpa*	1	0	0	2	7.24 ± 2.09
*Cab11*	*Maritima/adanensis*	37	0	0	0	7.15 ± 1.18
	*Maritima/macrocarpa*	37	0	0	0	9.74 ± 1.48
	*Adanensis/macrocarpa*	0	0	0	6	7.84 ± 2.61
*ITS*	*Maritima/adanensis*	3	1	0	0	2.70 ± 0.69
	*Maritima/macrocarpa*	3	0	0	7	12.86 ± 2.88
	*Adanensis/macrocarpa*	1	0	0	8	12.45 ± 3.53

Accordingly, *B. macrocarpa* represents a distinct genetic pool from *Beta vulgaris*, as it exhibits fixed differences at both genomic compartments with *B.v. maritima* and *B.v. adanensis*, while *B.v. maritima* and *B.v. adanensis* exhibit none.

The same pattern is less obvious when considering the nucleotide divergence among *Beta* section members (Dxy, Table 3). *B. macrocarpa* divergence with *B.v. maritima or B.v. adanen*sis at the chloroplastic level is comparable with the divergence among subspecies of *Beta vulgaris* at the chloroplastic level, but is higher at the nuclear loci, especially at the *ITS* locus with a level of nucleotide divergence that is 5 time as high between *B. macrocarpa* and *B. vulgaris* subspecies than the divergence among *Beta vulgaris* subspecies.

## 4 DISCUSSION

The present study aimed to survey the chloroplastic and nuclear genetic diversities of *Beta* species (*Beta* section) and explore the phylogenetic relationships among them.

Accordingly with former studies (Andrello et al., 2016, 2017; Kadereit et al., 2006; Letschert, 1993; Romeiras et al., 2016), *Beta macrocarpa* appeared to be a distinct monophyletic lineage from *Beta vulgaris* that comprised the two *subspecies B.v. maritima* and *B.v. adanensis*. The divergence date between *B. macrocarpa* and *B. vulgaris* has recently been estimated to be 1.4 Mya (Romeiras et al., 2016).

Within *Beta vulgaris*, the two subspecies *B.v. maritima* and *B.v. adanensis* were analysed on a representative geographical sampling. It must be noted that the two subspecies differ in their distribution and in their mating system. While *B.v. maritima* populations are found on a large geographical area, along the Atlantic coasts of Western Europe and the coasts of most Mediterranean countries, *B.v. adanensis* is restricted in the eastern part of the Mediterranean Basin (Aegean islands, Turkey, Syria and Iran). Therefore, the observation of a lower genetic diversity of *B.v. adanensis* when compared with *B.v. maritima* was expected. The low divergence between *B. vulgaris* subspecies can be explained by a recent differentiation of *B.v. adanensis* (indeed the *B. v. adanensis* lineage is not clearly defined and is moreover nested within the *B. v. maritima* clade) and/or contemporary gene flow between the subspecies, as populations of both subspecies can be found in close proximity. Controlled crosses are possible between the subspecies, confirming that reproductive barriers are limited (Hautekèete, 2001). In addition, differentiation of *B.v. adanensis* populations in situation of sympatry with *B.v. maritima* is most likely due to a transition from self‐incompatibility to self‐compatibility. Indeed, the present study suggests that *B. v. adanensis* reproduces mainly by selfing, as all analysed individuals were found homozygous at nuclear loci. This lack of heterozygosity could also be explained by the low level of diversity in the subspecies. However, the hypothesis of selfing conforms to the cytological pattern observed on self‐pollen germination in *B. v. adanensis* (Bruun et al., 1995). Further studies on a larger sampling and including a population level, in particular by contrasting parapatric versus allopatric situations, are necessary to estimate current gene flow that could occur between the subspecies, and the level of self‐fertilization in *B.v. adanensis*. The development of population genomic approaches thanks to next‐generation sequencing methodologies would be worthwhile to propose a demographic scenario of *B.v. adanensis* differentiation, measure the level introgression between both subspecies, the direction of gene flow, as well as the impact of the transition toward selfing on its genomic diversity (synonymous and nonsynonymous) as exemplified in *Capsella* or *Mimulus* (Brandvain, Kenney, Flagel, Coop, & Sweigart, 2014; Foxe et al., 2009).

In previous studies, *Beta macrocarpa* has been described as two cytotypes: one diploid cytotype widely distributed from Portugal to Turkey, along the Mediterranean Basin, and a tetraploid cytotype first found in the Canary Islands (Buttler, 1977) Earlier studies on this tetraploid cytotype have suggested a hybrid origin of the taxon between *B. v. maritima* and *B. macrocarpa*: (1) cytological observations revealed a complete diploidised meiosis as expected for an alloploid (Lange & de Bock, 1989), (2) genetic analyses on nuclear allozyme loci showed *B.v. maritima* and *B. macrocarpa* alleles‐like (Abe & Tsuda, 1987; Letschert, 1993), and (3) a maritima‐like chloroplastic haplotype was found in a Canarian individual (Kishima, Mikami, Hirai, Sigiura, & Kinoshita, 1987). Nevertheless, the occurrence of tetraploid individuals does not seem to be restricted to the Canary Islands as formerly believed: recent studies localized 4× individuals on another Macaronesian island, Santo Porto (Madeira Archipelago) (Leys et al., 2014) but also in continental populations from Southern Portugal (Castro et al., 2013). The present study confirms the hybrid origin of 4× *B. macrocarpa* from two Canary Islands (Gran Canaria and Tenerife): at the nuclear level each individual bears a maritima‐like allele and a macrocarpa‐like allele with the exception of ITS where only one allele, belonging to the *B.v. maritima* clade, was found. This is most likely due to concerted evolution as observed in allopolyploid Gossypium species (Wendel, Schnabel, & Seelanan, 1995), rice (Bao, Wendel, & Ge, 2010), or tobacco (in Bao et al., 2010).

At the chloroplastic level, both 4× Canarian individuals shared the same haplotype with *B.v. maritima* individuals. This suggests that the initial maternal parent of the hybrid was *B.v. maritima*, and thus *B. macrocarpa* was the pollen donor. The hybridization between self‐incompatible *B.v. maritima* and self‐compatible *B. macrocarpa* led to an alloploid species, described as self‐compatible in early studies (Buttler, 1977). Our results suggest that 4× individuals mainly reproduce by selfing, as we did not find any heterozygosity at the homeologous loci.

It must be noted that if the present study confirms the allopolyploid nature of 4× *B. macrocarpa* found in Canary Islands, it also shows for the first time that *B. macrocarpa* individuals found in the Canary Islands are not all tetraploid. Indeed, the individual from Fuerteventura (m2) exhibits all the genetic features of 2× continental *B. macrocarpa* at both chloroplastic and nuclear levels. This result raises the question of the occurrence of 2× *B. macrocarpa* populations in the Canary Islands where they were until now considered as absent. It remains to know the relative occurrence of the two forms in the Canary Islands as well as the geographical origin of 4× macrocarpa populations: whether the hybridization occurred in the islands or in the continent followed by long‐distance dispersal (Linder & Barker, 2014). Further studies are needed to describe the phenotypic characteristics and the ecological preferences of the different *macrocarpa* cytotypes in order to better distinguish them taxonomically but also to understand how the two types coexist in the Macaronesian archipelago and the adjacent regions.

## CONFLICT OF INTEREST

None declared.

## AUTHOR CONTRIBUTIONS

PT and JC conceived and designed the study. SV, LB, and A‐CH carried out the laboratory experiments. PT, SV, and CP carried out the analyses. PT and SV wrote the draft manuscript that was edited by CP and JC.
