# Supplementary material for: Chloroplastic and nuclear diversity of wild beets at a large geographical scale: Insights into the evolutionary history of the Beta section
Source: Ecol Evol. 2018 Feb 14;8(5):2890–900. doi: 10.1002/ece3.3774 (PMC5838056; doi:10.1002/ece3.3774)
Supplement: Supplementary file 1 [file ECE3-8-2890-s001.docx]

Figure S1: Haplotype network based on chloroplastic sequences using statistical parsimony generated by PopART (Leigh and Bryant 2015). For each node the name of only one accession is given (see table 1). However, when the node is shared by several accessions, accessions names are indicated below the haplotype network. Dashes on branches between nodes represent nucleotidic differences between haplotypes.


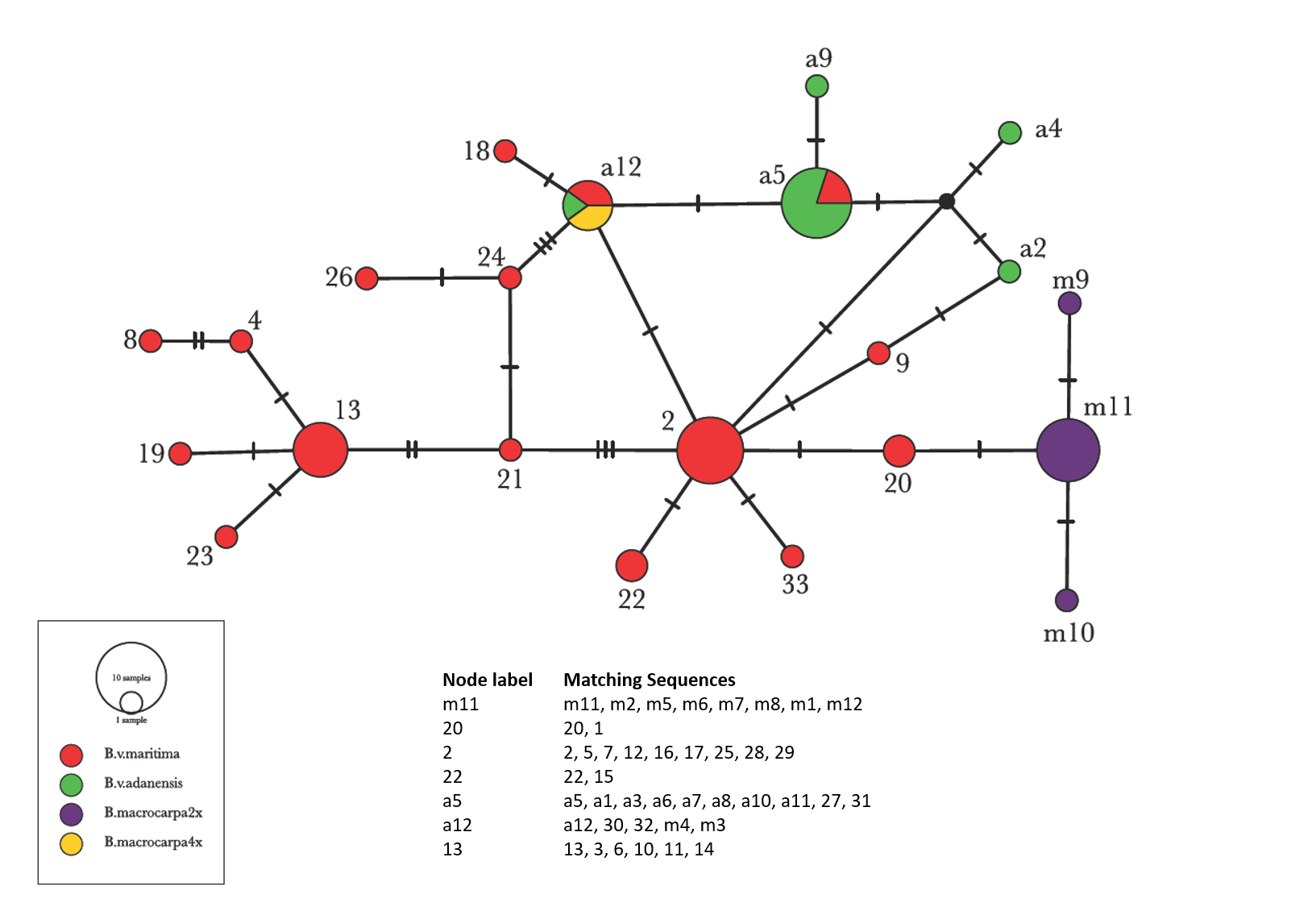


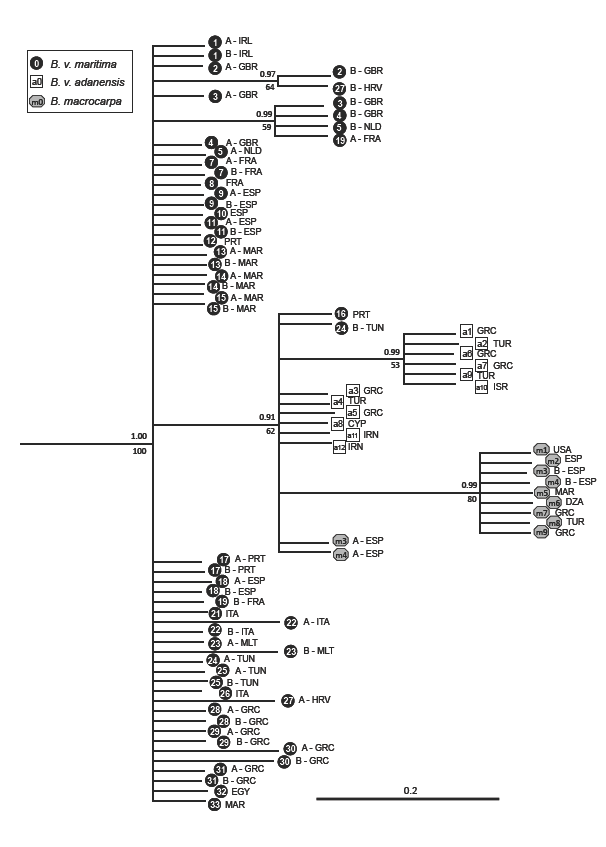


FigureS2: *Adh* phylogenetic tree as inferred by Bayesian analyses. Maximum likelihood analysis resulted in a close topology. Posterior probabilities and bootstrap percentages are indicated above and below the branches respectively. When individuals are heterozygous, alleles A and B are indicated. The *Corollinae* species used as outgroup are not shown on the figure for the purpose of clarity.


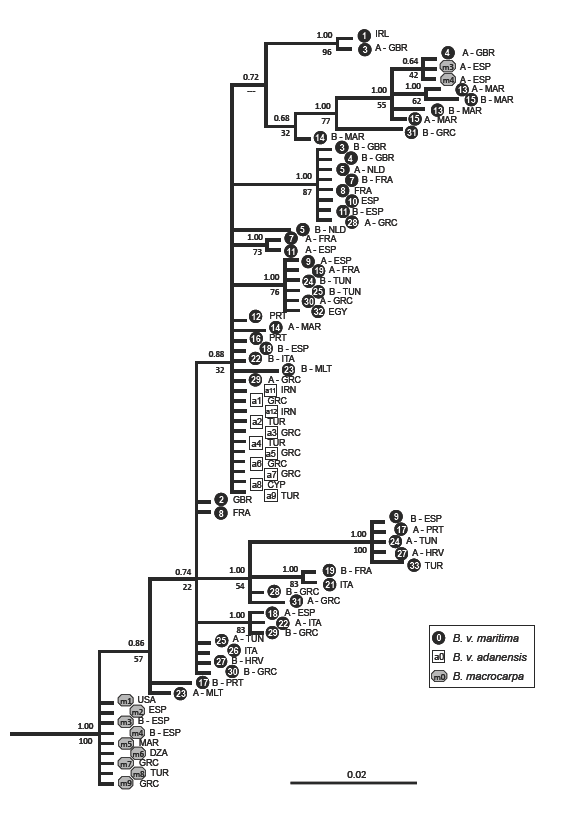


FigureS3: *Cab*11 phylogenetic tree as inferred by Bayesian analyses. Maximum likelihood analysis resulted in a close topology. Posterior probabilities and bootstrap percentages are indicated above and below the branches respectively. For incongruent nodes between Bayesian and ML topologies, dashes replace BP values. When individuals are heterozygous, alleles A and B are indicated The *Corollinae* species used as outgroup are not shown on the figure for the purpose of clarity.


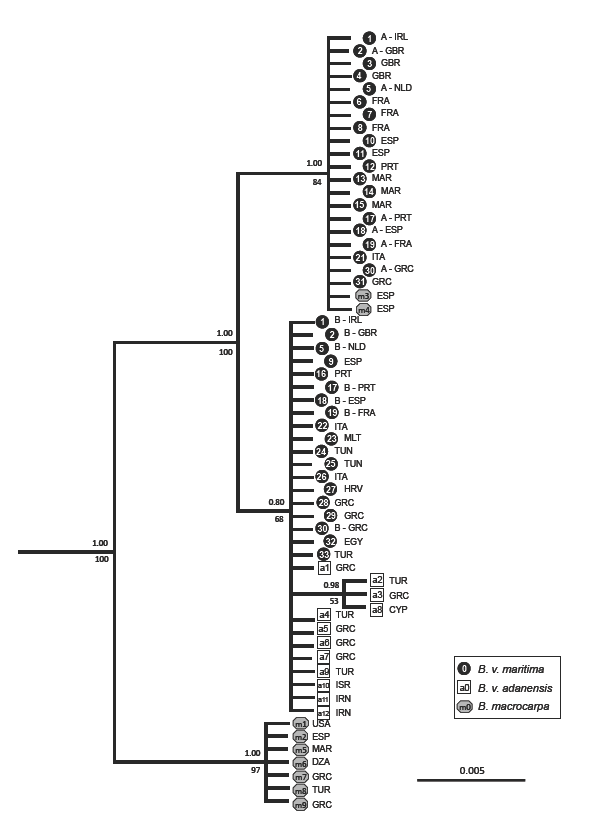


FigureS4: ITS phylogenetic tree as inferred by Bayesian analyses. Maximum likelihood analysis resulted in a close topology. Posterior probabilities and bootstrap percentages are indicated above and below the branches respectively. When individuals are heterozygous, alleles A and B are indicated. The *Corollinae* species used as outgroup are not shown on the figure for the purpose of clarity.
